# Characteristics and Trends Among Patients With Cardiovascular Disease Referred to Palliative Care

**DOI:** 10.1001/jamanetworkopen.2019.2375

**Published:** 2019-05-03

**Authors:** Haider J. Warraich, Steven P. Wolf, Robert J. Mentz, Joseph G. Rogers, Greg Samsa, Arif H. Kamal

**Affiliations:** 1 Department of Medicine, Duke University Medical Center, Durham, North Carolina; 2 Department of Biostatistics and Bioinformatics, Duke University, Durham, North Carolina; 3 Duke Clinical Research Institute, Durham, North Carolina; 4 Duke Cancer Institute, Durham, North Carolina

## Abstract

**Importance:**

Use of palliative care (PC) for patients with cardiovascular disease (CVD) has increased recently. However, it is unknown if patients are receiving earlier referrals to PC.

**Objective:**

To assess characteristics and trends of patients with CVD referred to PC.

**Design, Setting, and Participants:**

Cohort study in which analysis of data from the multicenter Quality Data Collection Tool for Palliative Care registry from January 2, 2015, through December 29, 2017, included patients with CVD 18 years or older referred to initial PC consultation who had a documented palliative performance score (PPS) .

**Exposures:**

Patients with CVD who presented for an initial PC visit.

**Main Outcomes and Measures:**

The primary outcome was PPS. Secondary outcomes included symptoms and end-of-life documentation.

**Results:**

Among 1801 patients (mean [SD] age, 77.7 [13.7] years) from 16 sites in the analysis, 875 (48.6%) were women and 1339 (74.3%) were white. A low PPS score (0%-30%), consistent with bedbound status, was recorded for 521 patients (28.9%), with no change through time. The most common moderate to severe symptoms were poor well-being, tiredness, anorexia, and dyspnea. Year of encounter was associated with improved symptoms of pain (odds ratio, 1.25; 95% CI, 1.05-1.50) and with constipation (odds ratio, 1.32; 95% CI, 1.03-1.69). No change through time was noted in other symptoms or end-of-life documentation. Although the proportion of referrals from general medicine increased from 43.2% (167 of 387) in 2015 to 52.9% (410 of 775) in 2017, the proportion of referrals from cardiologists decreased from 16.5% (64 of 387) in 2015 to 10.5% (81 of 775) in 2017. The proportion of patients referred to PC who were black decreased from 11.9% (46 of 387) in 2015 to 6.3% (49 of 775) in 2017. While 69.5% of all patients with CVD (1252 of 1801) had a primary diagnosis of heart failure, the proportion of non–heart failure CVD diagnoses, such as coronary artery disease and valvular heart disease, increased from 25.6% (99 of 387) in 2015 to 30.1% (233 of 775) in 2017.

**Conclusions and Relevance:**

Patients with CVD demonstrated significant symptom burden, and there was no evidence in the registry of change in the PPSs of patients with CVD referred to PC through time. Cardiologists provided comparatively fewer referrals to PC for patients with CVD, and this proportion decreased through time. The proportion of racial and ethnic minorities referred to PC was small and decreased through time. These findings reinforce the need for cardiologists to be more engaged with PC and consider referring appropriate patients with CVD sooner.

## Key Points

**Question:**

What are the characteristics of patients with cardiovascular disease who are referred to palliative care, and how have they changed through time?

**Findings:**

In this cohort study of Quality Data Collection Tool registry data, of 1801 patients with cardiovascular disease referred to palliative care from 2015 to 2017, 521 patients (28.9%) were bedbound, a proportion that did not change over time. Cardiologists referred a minority of patients with cardiovascular disease to palliative care.

**Meaning:**

These findings suggest that patients with cardiovascular disease have very advanced disease when referred to palliative care, and there was no evidence of improvement in functional status from referral through time.

## Teaser

This cohort study uses registry data to assess characteristics and trends among patients with cardiovascular disease referred to palliative care.

## Introduction

Palliative care (PC) is an interdisciplinary type of specialized care focused on improving or maintaining quality of life for patients with a serious illness and for their families.^1^ Hospice is a subset of PC for patients with more advanced serious illness and an expected survival of less than 6 months. Upstream from hospice care, PC does not have prognosis or disease severity requirements and aims to be included in care from the time of diagnosis onward. Its role is complementary to primary and specialty care clinicians, who may continue life-prolonging interventions while PC plays a supportive role for patients and their clinicians. Oftentimes, PC specialists are consulted to address complex symptom management issues, establish and document goals of care, and discuss psychosocial issues.

The American College of Cardiology^2^ and American Heart Association^3^ both recommend early incorporation of PC into routine cardiovascular care, recognizing the bevy of patient-centered benefits it produces.^4^ However, PC remains underused among patients with cardiovascular disease (CVD), particularly among patients with heart failure (HF), the terminal manifestation of most CVDs.^5,6^ Recent years have seen increased use of PC for noncancer diagnoses, including CVD.^7^ Although decades of advocacy calling attention to the palliative needs of patients with CVD is a likely driver of increased use of PC, it is unknown if patients are receiving earlier referrals to PC. Therefore, to better assess characteristics and trends of patients with CVD referred to PC, we performed an analysis of patients referred to specialty PC enrolled in the national Quality Data Collection Tool (QDACT)^8^ registry for PC.

## Methods

### Data Source

We included patients from the clinician-entered, patient-reported QDACT registry which includes specialty PC consultations across care settings throughout the serious illness trajectory data, from January 2, 2015, to December 29, 2017. Written informed consent was obtained, and the Duke University Medical Center Institutional Review Board approved this study. This study is reported using the Strengthening the Reporting of Observational Studies in Epidemiology (STROBE) reporting guideline.

The QDACT registry was founded in 2007 by the Global Palliative Care Quality Alliance to measure and improve quality of nonhospice PC delivery.^9,10,11^ A collaboration between academic medical centers and community-based PC practices, the QDACT registry is a centralized quality measurement registry that collects quality-of-care data from multiple practices.^8^ Practices in the Global Palliative Care Quality Alliance enroll their clinicians to enter point-of-care data during clinical encounters in a web-based registry portal. Practices use a random sampling technique to input patient data into the registry, minimizing selection bias and representing a broad sampling of clinicians, patients, diseases, and care settings. Data elements are mapped to several national quality measures, including those endorsed by the National Quality Forum, included in federal payment programs,^12^ produced or curated by national membership societies,^13,14^ and proposed by expert panels.^15,16,17,18^ Members of Global Palliative Care Quality Alliance join voluntarily, pay membership dues, and have minimum data submission expectations to participate. The membership roster^19^ includes private and academic-affiliated practices of all sizes, from small (1-3 clinicians) to large (>20 clinicians) systems across the United States.

### Patients

Patients were included in the analysis if they were aged 18 years or older, had documented palliative performance scores (PPSs), and had a first-time PC consultation for CVD. Demographic variables included age, sex, self-reported race/ethnicity, marital status, clinician-estimated prognosis, referral source, and type of CVD.

### Primary and Secondary Outcomes

The PPS is an assessment of performance status^20^ and was the primary outcome of our analysis. It ranges from 0 to 100, where a 10-point increase in PPS represents less assistance needed with activities of daily living. We categorized PPS as low (0-30, representing a patient who is bedridden and fully dependent), moderate (40-60, representing a patient who is frequently bedridden with moderate dependency), and high (70-100, representing a patient who is fully functional and independent) (eFigure in the Supplement).^21,22,23^ Secondary outcomes included Edmonton Symptom Assessment Scale score (range, 0-10, where a higher score represents a higher symptom burden), end-of-life code status, advance directive, and proxy documentation.

### Statistical Analysis

Descriptive statistics were calculated and described by year of encounter. We used χ^2^, Fisher exact, and Kruskal-Wallis tests as appropriate to estimate significance of differences by year of encounter. To test our primary hypothesis, if there was significant change in PPS by year of encounter, we fit a linear regression model with PPS as the dependent variable and year of encounter as the primary predictor. To avoid overfitting and collinearity, we only treated sex, race, and age as covariates in the model. For all secondary outcomes, logistic regression models were fit with year of encounter as the primary predictor and adjusted for the same covariates as our primary outcome. We modeled the odds of improvement in status from moderate to severe and none to mild in our secondary outcomes. We treated year of encounter as a continuous variable since our primary interest was determining if there was an increase in symptoms or advance care planning in later years. A 2-tailed *P* value less than .05 was considered statistically significant. All analyses were performed using SAS statistical software version 9.4 (SAS Institute).

## Results

Among 12 914 patients referred to PC, there were 1936 patients (15.0%) with CVD. After excluding 135 patients without documented PPSs, 1801 patients with CVD initially evaluated by PC specialists from 16 institutions from January 2, 2015, to December 29, 2017, were included (eTable 1 in the Supplement). We did not find evidence that the proportion of PPS documentation changed over the study period. The 1801 patients with CVD and with documented PPSs composed 13.9% of the 12 914 adult patients with documented PPSs treated by PC specialists, and this proportion did not change over time (eTable 2 in the Supplement). There were 1269 patients (70.5%) who were hospitalized, 265 patients (14.7%) at nursing facilities, 214 patients (11.9%) at home, and 32 patients (1.8%) at a clinic (21 patients had no reported location). The number of PC referrals per year for patients with CVD increased during the period, from 387 in 2015 to 775 in 2017 (Table 1). The mean (SD) age of patients was 77.7 (13.7) years. Of 1611 patients with data on code status, 790 patients (49.0%) opted to receive full resuscitation efforts at the start of the consultation, and 821 patients (51.0%) chose to have do-not-resuscitate or do-not-intervene orders. After PC consultation, 68.4% of patients (1168 of 1707) elected not to be resuscitated.

**Table 1.** Characteristics of Patients With Cardiovascular Disease Referred to Palliative Care

Characteristic	No. (%)				*P* Value
	2015 (n = 387)	2016 (n = 639)	2017 (n = 775)	Overall (N = 1801)	
Age, mean (SD) [range], y	76.8 (13.8) [23.3-101.3]	77.5 (13.5) [22.6-103.9]	78.3 (13.8) [20.2-102.5]	77.7 (13.7) [20.2-103.9]	.01
Sex					
Male	205 (53.0)	318 (49.8)	386 (49.8)	909 (50.5)	.36
Female	180 (46.5)	317 (49.6)	378 (48.8)	875 (48.6)	
NR	2 (0.5)	4 (0.6)	11 (1.4)	17 (0.9)	
Ethnicity					
Hispanic	8 (2.1)	11 (1.7)	19 (2.5)	38 (2.1)	<.001
Not Hispanic or Latino	358 (92.5)	536 (83.9)	611 (78.8)	1505 (83.6)	
NR	21 (5.4)	92 (14.4)	145 (18.7)	258 (14.3)	
Race					
White	300 (77.5)	507 (79.3)	532 (68.6)	1339 (74.3)	<.001
Black	46 (11.9)	45 (7.0)	49 (6.3)	140 (7.8)	
Other or unknown	18 (4.7)	27 (4.2)	62 (8.0)	107 (5.9)	
NR	23 (5.9)	60 (9.4)	132 (17.0)	215 (11.9)	
Marital status					
Not married, divorced, or other	212 (54.8)	311 (48.7)	399 (51.5)	922 (51.2)	<.001
Married	146 (37.7)	243 (38.0)	249 (32.1)	638 (35.4)	
NR	29 (7.5)	85 (13.3)	127 (16.4)	241 (13.4)	
Prognosis estimation					
Hours to weeks	57 (14.7)	128 (20.0)	124 (16.0)	309 (17.2)	.08
Hospice eligible (1-6 mo)	100 (25.8)	171 (26.8)	206 (26.6)	477 (26.5)	
Hospice ineligible (>6 mo)	130 (33.6)	163 (25.5)	236 (30.5)	529 (29.4)	
NR	100 (25.8)	177 (27.7)	209 (27.0)	486 (27.0)	
Referral source					
General medicine	167 (43.2)	319 (49.9)	410 (52.9)	896 (49.8)	<.001
Oncology	0	3 (0.5)	0	3 (0.2)	
Cardiology	64 (16.5)	79 (12.4)	81 (10.5)	224 (12.4)	
Neurology	1 (0.3)	3 (0.5)	9 (1.2)	13 (0.7)	
Pulmonary	3 (0.8)	7 (1.1)	5 (0.6)	15 (0.8)	
Critical care	12 (3.1)	44 (6.9)	51 (6.6)	107 (5.9)	
Other medical subspecialty	5 (1.3)	1 (0.2)	3 (0.4)	9 (0.5)	
Self (palliative medicine team)	2 (0.5)	2 (0.3)	6 (0.8)	10 (0.6)	
Surgical specialties	5 (1.3)	4 (0.6)	1 (0.1)	10 (0.6)	
Emergency department	0	1 (0.2)	0	1 (0.1)	
NR	128 (33.1)	176 (27.5)	209 (27.0)	513 (28.5)	
Cardiovascular disease type					.02
Heart failure	288 (74.4)	422 (66.0)	542 (69.9)	1252 (69.5)	
Other	99 (25.6)	217 (34.0)	233 (30.1)	549 (30.5)	
Coronary artery disease	51 (13.2)	98 (15.3)	114 (14.7)	263 (14.6)	.63
Valvular heart disease	18 (4.7)	27 (4.2)	30 (3.9)	75 (4.2)	.82
Peripheral vascular disease	8 (2.1)	16 (2.5)	21 (2.7)	45 (2.5)	.80

Abbreviation: NR, not reported.

There were temporal differences in race/ethnicity, marital status, Medicaid status, referral source, and CVD type during the course of the study. Although the proportion of patients referred from general medicine and critical care increased during the period from 43.2% (167 of 387) in 2015 to 52.9% (410 of 775) in 2017 and from 3.1% (12 of 387) in 2015 to 6.6% (51 of 775) in 2017, respectively, the proportion of patients referred from cardiologists decreased from 16.5% (64 of 387) in 2015 to 10.5% (81 of 775) in 2017. The proportion of black patients referred to PC decreased from 11.9% (46 of 387) in 2015 to 6.3% (49 of 775) in 2017. While most patients with CVD had a primary diagnosis of HF, the proportion of non-HF cardiovascular diagnoses, such as coronary artery disease, valvular heart disease, and peripheral vascular disease, increased from 25.6% (99 of 387) in 2015 to 34.0% (217 of 639) in 2016 and 30.1% (233 of 775) in 2017.

A total of 521 patients (28.9%) had low PPSs and no evidence of change during the study (Table 2). Among 1269 participants in an inpatient setting, 478 patients (37.7%) had low PPSs. The most common moderate to severe symptoms were poor well-being, tiredness, anorexia, and dyspnea (Figure). In our unadjusted analysis, there was a reduction in the proportion of patients with moderate to severe pain (22.8% [n = 74] in 2015 vs 16.5% [n = 90] in 2017; *P* = .047) and drowsiness (21.7% [n = 61] in 2015 vs 12.7% [n = 62] in 2017; *P* = .002) but an increase in the proportion of patients with poor well-being (37.0% [n = 51] in 2015 vs 53.5% [n = 230] in 2017; *P* < .001). After adjustment, we found no evidence of a difference in PPS by year of encounter (point estimate, −0.48 [95% CI, −1.55 to 0.59]). We found year of encounter was associated with reduction in moderate to severe pain or constipation (Table 3). No evidence of change through time was noted in code status, advanced directive, or health care proxy documentation.

**Table 2.** Performance Status, Symptoms, and Characteristics of Patients With Cardiovascular Disease Referred to Palliative Care

Symptom	No. (%)
Palliative performance status (N = 1801)	
Low (0%-30%)	521 (28.9)
Moderate (40%-60%)	1114 (61.9)
High (70%-100%)	166 (9.2)
Pain score (n = 1328)	
None or mild	1067 (80.3)
Moderate or severe	261 (19.7)
NR, No.	473
Dyspnea score (n = 1342)	
None or mild	968 (72.1)
Moderate or severe	374 (27.9)
NR, No.	459
Constipation (n = 1264)	
None or mild	1151 (91.1)
Moderate or severe	113 (8.9)
NR, No.	537
Tiredness (n = 1170)	
None or mild	582 (49.7)
Moderate or severe	588 (50.3)
NR, No.	631
Nausea (n = 1285)	
None or mild	1239 (96.4)
Moderate or severe	46 (3.6)
NR, No.	516
Depression (n = 1116)	
None or mild	983 (88.1)
Moderate or severe	133 (11.9)
NR, No.	685
Anxiety (n = 1217)	
None or mild	1049 (86.2)
Moderate or severe	168 (13.8)
NR, No.	584
Drowsiness (n = 1190)	
None or mild	986 (82.9)
Moderate or severe	204 (17.1)
NR, No.	611
Anorexia (n = 1173)	
None or mild	754 (64.3)
Moderate or severe	419 (35.7)
NR, No.	628
Well-being (n = 904)	
None or mild	434 (48.0)
Moderate or severe	470 (52.0)
NR, No.	897
Full code status (n = 1611)	
Full code	790 (49.0)
Do not resuscitate or intubate	821 (51.0)
NR, No.	190
Advance directive (n = 1414)	
No	436 (30.8)
Yes	978 (69.2)
NR, No.	387
Health care proxy (n = 1685)	
None	105 (6.2)
Yes	1580 (93.8)
NR, No.	116

Abbreviation: NR, not reported.

**Figure.**
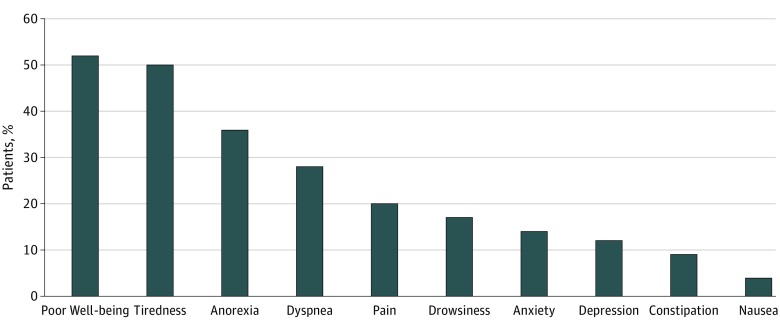
Proportion of Patients With Cardiovascular Disease Referred to Palliative Care With Moderate to Severe Symptoms

**Table 3.** Adjusted Association of Year of Palliative Care Referral With Change in Moderate to Severe Symptoms and With Advance Care Planning

Outcome	Sample, No.	Odds Ratio (95% CI)^a^
Pain	1328	1.25 (1.05-1.50)
Dyspnea	1342	1.04 (0.89-1.21)
Constipation	1264	1.32 (1.03-1.69)
Nausea	1285	1.24 (0.85-1.80)
Resuscitation	1611	1.06 (0.92-1.21)
Health care proxy	1685	1.22 (0.95-1.57)
Advance directive	1414	1.09 (0.94-1.27)

^a^ Adjusted for age, race, and sex.

There were several differences between patients referred to PC consultation from general medicine vs cardiology, the 2 most common referring specialties (Table 4). Compared with patients referred from general medicine (n = 896), patients referred from cardiologists (n = 224) were on average younger (mean [SD] age, 81.3 [11.7] years vs 74.0 [14.4] years) and had higher proportions of nonwhite race (10.0% [n = 90] vs 22.8% [n = 51]), Hispanic ethnicity (0.2% [n = 2] vs 4.9% [n = 11]), and HF (68.4% [n = 613] vs 77.2% [n = 173]) . However, mean (SD) PPS was similar in patients referred from general medicine (43.8 [17.7]) and from cardiology (45.4 [13.5]) (*P* = .25).

**Table 4.** Characteristics of Patients With Cardiovascular Disease Referred to Palliative Care by General Medicine and Cardiology Specialties

Characteristic	No. (%)			*P* Value
	General Medicine (n = 896)	Cardiology (n = 224)	Overall (n = 1120)	
Year of referral				
2015	167 (18.6)	64 (28.6)	231 (20.6)	<.001
2016	319 (35.6)	79 (35.3)	398 (35.5)	
2017	410 (45.8)	81 (36.2)	491 (43.8)	
Age, mean (SD) [range], y	81.3 (11.7) [22.6-101.9]	74.0 (14.4) [25.7-99.7]	79.8 (12.6) [22.6-101.9]	<.001
Sex				
Male	389 (43.4)	128 (57.1)	517 (46.2)	<.001
Female	499 (55.7)	96 (42.9)	595 (53.1)	
NR	8 (0.9)	0	8 (0.7)	
Ethnicity				
Hispanic	2 (0.2)	11 (4.9)	13 (1.2)	<.001
Not Hispanic or Latino	764 (85.3)	204 (91.1)	968 (86.4)	
NR	130 (14.5)	9 (4.0)	139 (12.4)	
Race				
White	701 (78.2)	162 (72.3)	863 (77.1)	<.001
Black	46 (5.1)	33 (14.7)	79 (7.1)	
Other or unknown	44 (4.9)	18 (8.0)	62 (5.5)	
NR	105 (11.7)	11 (4.9)	116 (10.4)	
Marital status				
Not married, divorced, or other	518 (57.8)	111 (49.6)	629 (56.2)	<.001
Married	275 (30.7)	102 (45.5)	377 (33.7)	
NR	103 (11.5)	11 (4.9)	114 (10.2)	
Medicaid				
No	311 (34.7)	102 (45.5)	413 (36.9)	<.001
Yes	107 (11.9)	35 (15.6)	142 (12.7)	
NR	478 (53.3)	87 (38.8)	565 (50.4)	
Prognosis estimation				
Hours to weeks	161 (18.0)	16 (7.1)	177 (15.8)	<.001
Hospice eligible (1-6 mo)	245 (27.3)	61 (27.2)	306 (27.3)	
Hospice ineligible (>6 mo)	321 (35.8)	37 (16.5)	358 (32.0)	
NR	169 (18.9)	110 (49.1)	279 (24.9)	
Cardiovascular disease type				
Heart failure	613 (68.4)	173 (77.2)	786 (70.2)	<.001
Other	283 (31.6)	51 (22.8)	334 (29.8)	

Abbreviation: NR, not reported.

## Discussion

These data provide a comprehensive overview of patients with CVD referred to PC from 16 diverse sites across the United States from 2015 through 2017. Almost one-third of the patient population was bedbound at initial referral, with no evidence of change noted during the period. The proportion of PC referrals from cardiologists decreased from 16.5% of referrals in 2015 to 10.5% of referrals in 2017. The proportion of black patients with CVD referred to PC also decreased from 11.9% in 2015 to 6.3% in 2017. Most consultations occurred in the inpatient setting, and HF accounted for 69.5% of patients with CVD, although other diagnoses, such as ischemic heart disease and valvular heart disease, have become more common. Additionally, although HF accounted for most patients with CVD referred to PC, there was an increase in the proportion of non-HF CVD diagnoses during our study.

These data provide further insights into trends in use of specialty PC in patients with CVD, in addition to patients with HF, from a large and diverse registry from academic and community settings, rural and urban areas, and inpatient and outpatient settings. While patients in hospice are better characterized, with a 2018 study^7^ showing that hospice referrals for patients with CVD have increased over time but still lag considerably behind other conditions, such as cancer, less is known about PC consultations. Data from the Veterans Affairs population^24^ and a privately insured population^25^ suggest that PC use has increased modestly through time for patients with HF. In our population, we did not observe an increase in the proportion of patients with CVD referred to PC during the 3 years in our registry.

Our data suggest that patients with CVD are referred to PC fairly late in their disease trajectory. More than one-quarter of our patients with CVD had a PPS of 0% to 30% at time of initial PC referral compared with about 1 in 10 patients with cancer with similar PPSs from a 2017 QDACT analysis.^22^ Patients in this PPS range are bedbound, are drowsy or comatose, require total care, and were found in a 2009 study^26^ of patients in hospice with and without cancer to have a median survival 0 to 5 days. In addition, we saw substantial proportions of patients reporting moderate to severe symptoms, including poor well-being (52.0%), tiredness (50.3%), anorexia (35.7%), dyspnea (27.9%), and pain (19.7%). Although PC can be of particular value for patients in the last days of life, more value could be gained by patients if they were able to access PC earlier. Given that Medicare beneficiaries admitted with HF have a median survival of 2 years, hospitalization might be an opportunity to introduce PC to patients with HF.^27^ Inpatients in our analysis had a higher proportion of low PPSs. Further work is needed to develop clinical guidelines that provide tailored PC to patients with advanced CVD.^28^ Most of our patient population had an advance directive completed and a health care proxy identified at the time of referral. These proportions are much higher than in the general population and represent improvements in primary PC delivered by the referring teams.^29^

Our data demonstrate that patients with CVD are referred to PC largely from general medicine. Cardiologists accounted for a declining proportion of referrals to PC. The implications for this finding are unclear; however, a 2016 study^30^ showed that many cardiologists are not comfortable discussing PC or prognoses with patients with HF, and this may be one reason driving this trend. Patients referred by cardiologists had a younger mean age and a higher proportion of HF, although they had similar PPSs to patients referred from general medicine. Efforts should be made to further engage all clinicians, including cardiologists, in providing early and appropriate access to PC for their patients.

### Limitations

Our study has several limitations. Given the retrospective, passive, and observational nature of this analysis, our discussion is limited to associations. Among our secondary outcomes, there were considerable data that were not reported. However, since these outcomes were not of primary interest, we decided against using their presence as an inclusion criterion. Additionally, generalizability of the study is limited to the United States, despite enrollment from 16 diverse sites.

## Conclusions

In conclusion, we did not find evidence to reject the hypothesis that performance status of patients with CVD referred to PC remained constant over time. Cardiologists provided relatively few referrals to PC for patients with CVD, and this proportion decreased through time. Proportions of racial and ethnic minorities referred to PC decreased through time. Opportunities for PC specialists to build collaborative approaches to provide early PC for patients with CVD should be studied.

## Notes

Supplement. **eFigure.** Patient Characteristics and Survival by Palliative Performance Score **eTable 1.** Enrollment of Patients by Year and Site **eTable 2.** Distribution of Patients Included and Excluded in Analysis
