# Supplementary material for: Characteristics and Trends Among Patients With Cardiovascular Disease Referred to Palliative Care
Source: JAMA Netw Open. 2019 May 3;2(5):e192375. doi: 10.1001/jamanetworkopen.2019.2375 (PMC6503632; doi:10.1001/jamanetworkopen.2019.2375)
Supplement: Supplement. — eFigure. Patient Characteristics and Survival by Palliative Performance Score eTable 1. Enrollment of Patients by Year and Site eTable 2. Distribution of Patients Included and Excluded in Analysis [file jamanetwopen-2-e192375-s001.pdf]

Supplementary Online Content

Warraich HJ, Wolf SP, Mentz RJ, Rogers JG, Samsa G, Kamal AH. Characteristics and trends among patients with cardiovascular disease referred to palliative care. *JAMA Netw Open*. 2019;2(5):e192375. doi:10.1001/jamanetworkopen.2019.2375

eFigure. Patient Characteristics and Survival by Palliative Perfomance Score
eTable 1. Enrollment of Patients by Year and Site
eTable 2. Distribution of Patients Included and Excluded in Analysis

This supplementary material has been provided by the authors to give readers additional information about their work.

© 2019 Warraich HJ et al. *JAMA Network Open.*

eFigure. Patient Characteristics and Survival by Palliative Perfomance Score

Patient Characteristics & Survival by Palliative Performance Score (PPS)

	High 100%-70%	Moderate 60%-40%	Low 30%-0%
Ambulation	Full-Reduced	Reduced-Mainly in Bed	Bed Bound-Death
Activity Level	Normal-Inability to do Job or Work	Cannot do Housework-Cant do any Work	Cannot do any Work
Self-Care	Full	Occasional Assistance-Mainly Assistance	Total Care
Intake	Normal-Reduced	Normal-Reduced	Reduced-Mouth Care Only
Level of Consciousness	Full	Full or Drowsy or Confused	Drowsy or Coma
Median Survival in days (Lau et al, J Pain Symptom Manage, 2009)	60 (PPS 70%)	14-45	0-5

© 2019 Warraich HJ et al. *JAMA Network Open.*

eTable 1: Enrollment of Patients by Year and Site.				
	2015 (N=387)	2016 (N=639)	2017 (N=775)	Total (N=1801)
**Institutions**				
Missing	2 (0.5%)	0 (0.0%)	0 (0.0%)	2 (0.1%)
Adventist Oregon	4 (1.0%)	19 (3.0%)	0 (0.0%)	23 (1.3%)
Butler Health	7 (1.8%)	82 (12.8%)	95 (12.3%)	184 (10.2%)
Capital Caring	1 (0.3%)	0 (0.0%)	0 (0.0%)	1 (0.1%)
Catawba	36 (9.3%)	33 (5.2%)	47 (6.1%)	116 (6.4%)
Four Seasons	202 (52.2%)	181 (28.3%)	243 (31.4%)	626 (34.8%)
Greenville Health Systems (GHS)	0 (0.0%)	40 (6.3%)	46 (5.9%)	86 (4.8%)
Hospice Care of SC	0 (0.0%)	7 (1.1%)	17 (2.2%)	24 (1.3%)
ICS	10 (2.6%)	0 (0.0%)	0 (0.0%)	10 (0.6%)
Main Line Health	0 (0.0%)	1 (0.2%)	9 (1.2%)	10 (0.6%)
Mayo	1 (0.3%)	0 (0.0%)	0 (0.0%)	1 (0.1%)
Mission Hospital	0 (0.0%)	112 (17.5%)	117 (15.1%)	229 (12.7%)
Partners in Care	0 (0.0%)	5 (0.8%)	1 (0.1%)	6 (0.3%)
SRMC	25 (6.5%)	39 (6.1%)	51 (6.6%)	115 (6.4%)
Transitions Life Care	1 (0.3%)	7 (1.1%)	4 (0.5%)	12 (0.7%)
UC Denver	16 (4.1%)	98 (15.3%)	129 (16.6%)	243 (13.5%)
UNC	82 (21.2%)	15 (2.3%)	16 (2.1%)	113 (6.3%)

© 2019 Warraich HJ et al. *JAMA Network Open.*

eTable 2: Distribution of Patients Included and Excluded in Analysis.							
	**2015 Excluded (N=2922)**	**2015 Included (N=387)**	**2016 Excluded (N=6112)**	**2016 Included (N=639)**	**2017 Excluded (N=6127)**	**2017 Included (N=775)**	**Total (N=16962)**
**PPS**							
N	2361	387	4298	639	4454	775	12914
Mean (SD)	46.5 (18.7)	44.8 (17.0)	42.9 (19.9)	41.7 (18.5)	42.0 (19.1)	43.2 (17.5)	43.3 (19.2)
Median	50.0	50.0	40.0	40.0	40.0	40.0	40.0
Q1, Q3	40.0, 60.0	40.0, 50.0	30.0, 60.0	30.0, 50.0	30.0, 50.0	30.0, 50.0	30.0, 60.0
Range	(0.0-100.0)	(0.0-90.0)	(0.0-100.0)	(0.0-90.0)	(0.0-100.0)	(0.0-100.0)	(0.0-100.0)
**PPS**							
Missing	561 (19.2%)	0 (0.0%)	1814 (29.7%)	0 (0.0%)	1673 (27.3%)	0 (0.0%)	4048 (23.9%)
0	3 (0.1%)	1 (0.3%)	17 (0.3%)	3 (0.5%)	16 (0.3%)	1 (0.1%)	41 (0.2%)
10	169 (5.8%)	28 (7.2%)	442 (7.2%)	77 (12.1%)	464 (7.6%)	69 (8.9%)	1249 (7.4%)
20	141 (4.8%)	21 (5.4%)	360 (5.9%)	45 (7.0%)	328 (5.4%)	38 (4.9%)	933 (5.5%)
30	272 (9.3%)	46 (11.9%)	674 (11.0%)	79 (12.4%)	770 (12.6%)	113 (14.6%)	1954 (11.5%)
40	463 (15.8%)	77 (19.9%)	891 (14.6%)	139 (21.8%)	964 (15.7%)	183 (23.6%)	2717 (16.0%)
50	593 (20.3%)	120 (31.0%)	764 (12.5%)	156 (24.4%)	880 (14.4%)	212 (27.4%)	2725 (16.1%)
60	342 (11.7%)	59 (15.2%)	504 (8.2%)	81 (12.7%)	483 (7.9%)	87 (11.2%)	1556 (9.2%)
70	228 (7.8%)	20 (5.2%)	403 (6.6%)	34 (5.3%)	332 (5.4%)	41 (5.3%)	1058 (6.2%)
80	107 (3.7%)	11 (2.8%)	177 (2.9%)	23 (3.6%)	151 (2.5%)	21 (2.7%)	490 (2.9%)
90	38 (1.3%)	4 (1.0%)	56 (0.9%)	2 (0.3%)	55 (0.9%)	8 (1.0%)	163 (1.0%)
100	5 (0.2%)	0 (0.0%)	10 (0.2%)	0 (0.0%)	11 (0.2%)	2 (0.3%)	28 (0.2%)
Abbreviations: PPS – palliative performance score, SD – standard deviation							

© 2019 Warraich HJ et al. *JAMA Network Open.*
